# Ultrasonic cleaning is effective in removing carbonized clots and tissue from the insulation‐tipped diathermic knife‐2

**DOI:** 10.1002/deo2.101

**Published:** 2022-03-06

**Authors:** Kei Murakami, Daizen Hirata, Kengo Haraguchi, Noriko Arai, Koji Inoue, Yuka Miyazaki, Kimihiro Funase, Tadao Nakashige, Akira Teramoto, Mineo Iwatate, Santa Hattori, Mikio Fujita, Wataru Sano, Yasushi Sano

**Affiliations:** ^1^ Gastrointestinal Center and Institute of Minimally‐invasive Endoscopic Care Sano Hospital Hyogo Japan; ^2^ Department of Gastroenterology and Hepatology Kindai University Osaka Japan; ^3^ Gastrointestinal Center Kishiwada Tokushukai Hospital Osaka Japan; ^4^ Kansai Medical University Osaka Japan

**Keywords:** carbonized clots and tissue, endoscopic device, endoscopic submucosal dissection, IT knife‐2, ultrasonic cleaning

## Abstract

**Objectives:**

Since carbonized clots and tissue (debris) tend to adhere firmly to the tip of the endoscopic submucosal dissection (ESD) knife as the procedure proceeds, manual removing the firm debris is often challenging and time‐consuming. Recently, effective ultrasonic cleaning for other medical devices has been reported. The aim of the present study was to clarify whether ultrasonic cleaning is effective in removing the debris on the insulation‐tipped diathermic (IT) knife‐2.

**Methods:**

This study was an ex‐vivo experimental randomized study. A total of 40 IT knife‐2 knives with debris on their tip surfaces were prepared and randomly assigned to two groups (Group A and Group B). The knives in Group A were cleaned using the conventional scrubbing method for 30 s (conventional cleaning method), while those in Group B were cleaned using a combined method of scrubbing for 20 s and ultrasonic cleaning for 10 s (combined ultrasonic cleaning method). The tip electrode of the knife after cleaning was photographed under a microscope (40x). The 40 images of the knives were evaluated by independent three endoscopists and two clinical engineers using the five‐step evaluation criteria ranging from cleaning score 1 (dirty) to 5 (clean).

**Results:**

The mean cleaning score of 3.78 (range: 2.33–4.67) in Group B was significantly higher than that of 1.68 (range: 1.00–2.83) in Group A.

**Conclusions:**

The combined ultrasonic cleaning method could remove debris adhering to the IT knife‐2 more effectively than the conventional cleaning method. Ultrasonic cleaning may be applied for real‐world ESD.

## INTRODUCTION

In recent years, endoscopic submucosal dissection (ESD) has been performed to treat early‐stage gastrointestinal cancer.^1^, ^2^ During ESD, carbonized clots and tissue (debris) tend to adhere to the tip of the ESD knife,^3^ which suppresses the current flow to the tip, resulting in its reduced incision and coagulation capabilities. Therefore, the adherent debris needs to be removed frequently during the procedure, and the improvement of the debris removal technology is one of the important issues for safe and efficient ESD.

In many facilities, the debris is usually removed by scrubbing the tip of the knife with gauze soaked in saline or pronase. However, it is exceedingly difficult to remove the debris quickly and sufficiently using this method, and the residue is often left behind. Moreover, there is a risk of damaging the tip by rubbing too hard. For these reasons, an easy and efficient cleaning method is required.

Ultrasonic cleaning has long been used for accessories, eyeglasses, industrial equipment, and reportedly in recent years, for medical devices such as dental implants.^5^, ^6^ Thus, in order to clarify whether ultrasonic cleaning is also effective in removing the debris on ESD knives, a comparative study was conducted on the insulation‐tipped diathermic (IT) knife‐2 (Olympus Co., Tokyo, Japan).

## METHODS

**FIGURE 1:**
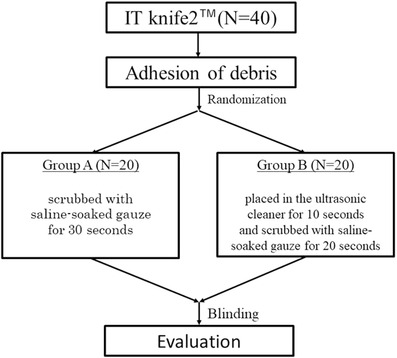
Flowchart of the study design

This study was an ex‐vivo experimental randomized study (Figure 1).

### Materials

**FIGURE 2:**
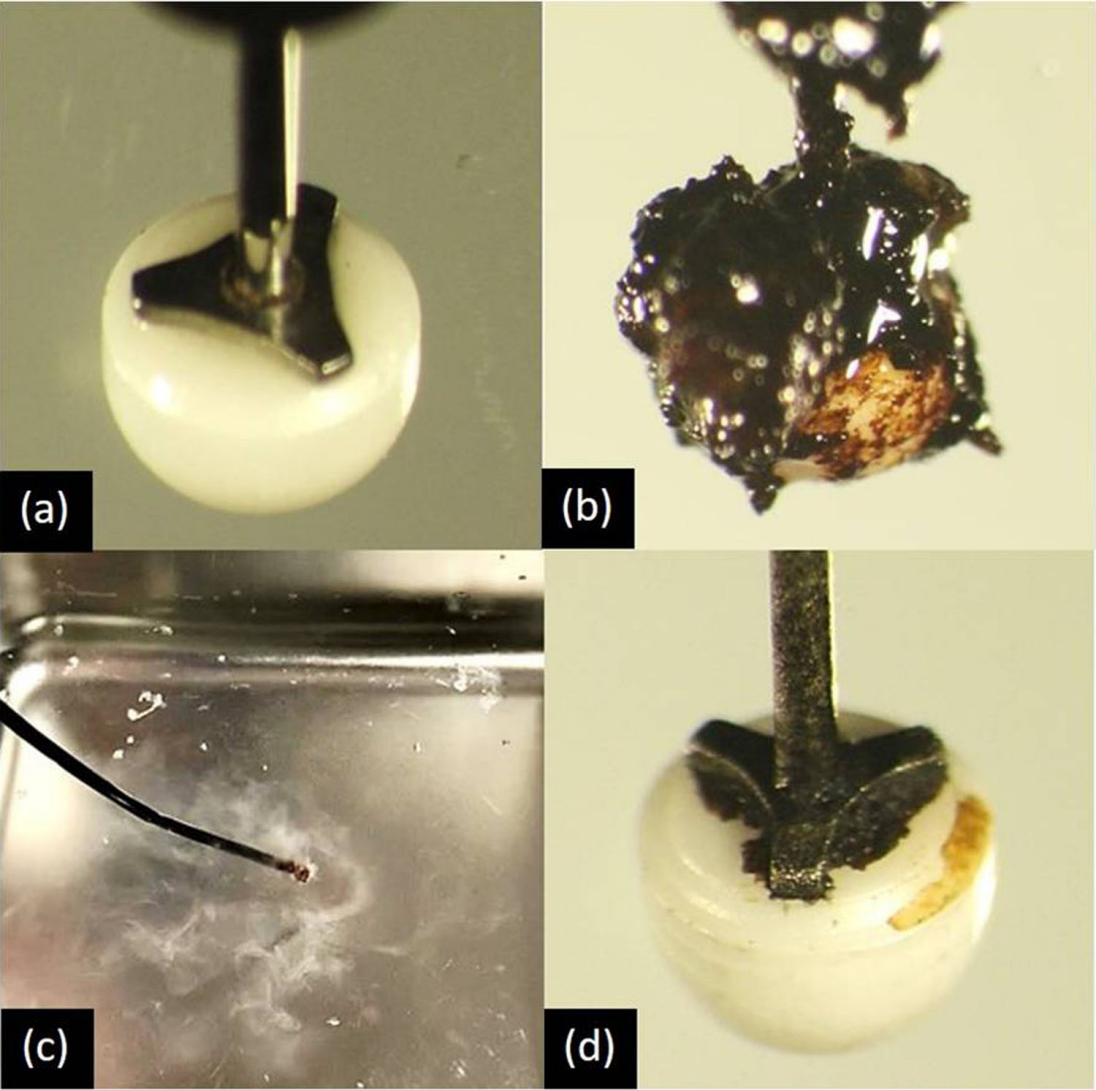
Changes in endoscopic submucosal dissection (ESD) knives. (a) Before use, (b) debris adhered, (c) ultrasonic cleaning, and (d) after combined ultrasonic cleaning

A total of 40 IT knife‐2 knives with debris on their tip surfaces were prepared as samples for use in this study. The preparation method of these knives was as follows:

1. Two milliliters of human blood collected from a healthy volunteer was spread on thin slices of pork.
2. The IT knife‐2 (Figure 2a) was connected to the VIO300D (ERBE Co., Tübingen, Germany) and set to “SWIFT COAG, Effect 5–100 W”.
3. While energizing with the above settings, the tip of the knife was brought into contact with the pork and was slid for over 3 s at a distance of 2 cm. This operation was performed a total of five times to adhere to a sufficient amount of debris on the tip electrode part. A total of 40 knives with debris (Figure 2b) were prepared for this randomized trial.

### Study design

The prepared 40 knives with debris were randomly assigned to two groups (Groups A and B). The knives in Group A were cleaned using the conventional scrubbing method (conventional cleaning method), while those in Group B were cleaned using a combined method of scrubbing and ultrasonic cleaning (combined ultrasonic cleaning method). Based on our experience that the time available for the device cleaning during ESD was approximately 30 s at a time, the cleaning time was set to 30 s for both groups in this study. In Group A, the tip electrode of the knife was scrubbed with saline‐soaked gauze for 30 s. In Group B, the knife was placed in the ultrasonic cleaner for 10 s (Figure 2c), and then the tip electrode was scrubbed with gauze soaked in saline solution for 20 s. The USC‐1 ultrasonic cleaner (AS ONE Co., Osaka, Japan) with a frequency of 40 kHz and a cleaning solution made of RO pure water were used in this study. An actual example of ultrasonic cleaning is shown in the Supporting Information video. These methods are in accordance with the Clinical Trials Act and have been reviewed and approved by the Research Ethics Review Committee (Case No: 201911‐02). This study protocol adhered to the principles of the Declaration of Helsinki.

### Outcome measure

Each cleaned tip electrode of the 40 IT knife‐2 composed of 20 Group A and 20 Group B was photographed under a microscope (40x) (Figure 2d). The 40 images of the knives were randomly assigned using the single‐blind method and evaluated by independent three endoscopists and two clinical engineers in accordance with the five‐step evaluation criteria ranging from cleaning score 1 (dirty) to 5 (clean) (Table 1). The primary outcome measure was the difference in mean cleaning scores between Groups A and B.

**TABLE 1:** Evaluation criteria for cleaning efficiency (cleaning score)

**Score**	**Schema**	**Image**	**Criteria**
1	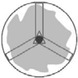	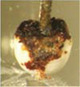	Almost all tip is covered with debris.
2	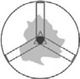	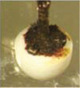	The outer periphery of the tip is visible. More than half of the tip is covered with debris.
3	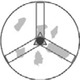	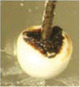	Less than half of the tip is covered with debris. Debris is visible on the insulator area and the metal area.
4	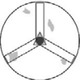	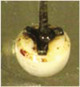	The insulator area is almost clean. Debris is visible mainly on the metal area.
5	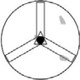	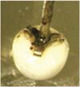	Almost all of the tip is clean.

Five‐step evaluation criteria ranging from cleaning score 1 (dirty) to 5 (clean) for IT knife‐2

### Statistical analysis

Mann‐Whitney's U test was used to compare between Group A and B. A *p*‐value < 0.05 was considered statistically significant. R Version 4. 0. 0 (R Core Team (2020), Vienna, Austria) was used for the statistical analysis in this study.

## RESULTS

**FIGURE 3:**
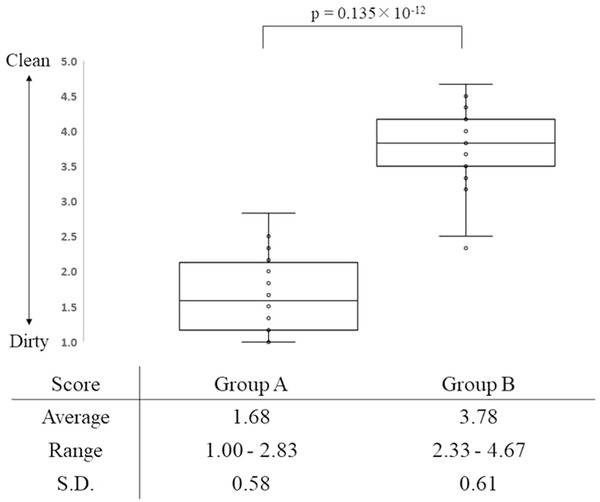
Cleaning score. The mean cleaning score of Group A was 1.68, with a range of 1.00–2.83 and that of Group B was 3.78, with a range of 2.33–4.67. The *p*‐value was 0.135 × 10^−12^

The cleaning efficiency could be evaluated in all 40 IT knife‐2 knives without any damage. The mean cleaning score of Group A (conventional cleaning method) was 1.68, with a range of 1.00–2.83, and that of Group B (combined ultrasonic cleaning method) was 3.78, with a range of 2.33–4.67 (Figure 3). The *p*‐value was 0.135 × 10^−12^, indicating a statistically significant difference between the two groups.

## DISCUSSION

To the best of our knowledge, this study was the first ex‐vivo experimental study of an ultrasonic cleaning machine for an endoscopic instrument. In the present study, ultrasonic cleaning was introduced in addition to conventional physical scrubbing. The combined ultrasonic cleaning method was able to more effectively remove firm debris adhering to the IT knife‐2 than the conventional cleaning method. Furthermore, the principle of such debris adhesion is considered to be the same for devices other than the IT knife‐2, and the combined ultrasonic cleaning method is expected to be effective in removing firm debris for other devices.

In ultrasonic cleaning, ultrasonic waves in the range of 20–100 kHz are irradiated into the liquid, and the resulting shock waves generated by the cavitation phenomenon peel off foreign substances from the object. These shock waves are also reported to be effective in disrupting bacterial cell membranes and reducing the number of bacteria.^7^, ^8^ This is called the “cavitation effect,” which is stronger at lower frequencies and weaker at higher frequencies. In our pre‐study experiments, it took about 60–90 s for the conventional method and 30–40 s for ultrasonic cleaning (40 kHz) to achieve a cleaning score of 5 for almost all models. This suggests ultrasonic cleaning has a higher ability to clean the debris and reduce cleaning time than the conventional method.

Potential concerns of the induction of ultrasonic cleaning include damage to the device, cost performance, protection from infection. None of the knives used in this trial were damaged during the procedure, and an additional 30 min of ultrasonic cleaning of those knives did not reveal any obvious damage. However, if soft metals such as aluminum and silver are subjected to a strong cavitation effect for a long period of time, their surfaces may be damaged. Therefore, it may be better to change the frequency and time settings depending on the material to be cleaned. The prices of ultrasonic cleaners are coming down, and we can buy a good one for around 80–120 USD. Considering the running cost is also very inexpensive with a small amount of distilled water and electricity, ultrasonic cleaning can be cost‐effective. Some doctors might be concerned that repeating the use of ultrasonic cleaning can cause infection. Although we used an integrated type of ultrasonic cleaner in the study, its sterilization operation was complex, removable type ultrasonic cleaners are available now. Since the containers can be replaced for each patient easily and autoclaved or treated with sterile gas after every procedure, the risk of infection is not a matter.

The present study has limitations. First, this was a single‐center ex‐vivo study. Further studies in a real‐world setting are needed in the future. Second, during the preparation process of the knives, the fact that the person scrubbing the knife would know which group the knife belonged to could be a bias in this study. However, in order to minimize the bias, we tried to minimize differences in the intensity and speed of scrubbing. Therefore, we believe that this will have little effect on the results. Third, we made debris by coagulating 2 ml of human blood on the pork for 3 s, repeating it five times. Longer cutting duration and the use of pork may affect our result compared with the situation in actual ESD.

In conclusion, the results from this study showed that the combined ultrasonic cleaning method could remove debris adhering to the IT knife‐2 more effectively than the conventional cleaning method. The results also suggest that this combined ultrasonic cleaning method can be safely and efficiently applied to ESD in clinical practice.

## FUNDING INFORMATION

None

## CONFLICT OF INTEREST

The authors declare no conflict of interest.

## Supporting information


**Supplementary Video**: The procedure of ultrasonic cleaning in removing carbonized clots and tissue from IT knife‐2 knives
